# Predictive biomarker discovery through the parallel integration of clinical trial and functional genomics datasets

**DOI:** 10.1186/gm174

**Published:** 2010-08-11

**Authors:** Charles Swanton, James M Larkin, Marco Gerlinger, Aron C Eklund, Michael Howell, Gordon Stamp, Julian Downward, Martin Gore, P Andrew Futreal, Bernard Escudier, Fabrice Andre, Laurence Albiges, Benoit Beuselinck, Stephane Oudard, Jens Hoffmann, Balázs Gyorffy, Chris J Torrance, Karen A Boehme, Hansjuergen Volkmer, Luisella Toschi, Barbara Nicke, Marlene Beck, Zoltan Szallasi

**Affiliations:** 1 Cancer Research UK London Research Institute, 44 Lincoln's Inn Fields, London, WC2A 3PX, UK; 2 Department of Medicine, Royal Marsden Hospital, Fulham Road, London, SW3 6JJ, UK; 3 Institute of Cancer, Barts and the London School of Medicine and Dentistry, Charterhouse Square, London, EC1M 6BQ, UK; 4 Center for Biological Sequence Analysis, Technical University of Denmark, DK-2800 Lyngby, Denmark; 5 Wellcome Trust Sanger Institute, Wellcome Trust Genome Campus, Hinxton, Cambridge, CB10 1SA, UK; 6 Institut Gustave Roussy, 114 rue Edouard Vaillant, 94805 Villejuif, France; 7 Hôpital Européen Georges Pompidou, 20 Rue Leblanc, 75015 Paris, France; 8 EPO-Berlin GmbH, Robert-Rössle-Str.10, 13125 Berlin, Germany; 9 Joint Research Laboratory of the Hungarian Academy of Sciences and the Semmelweis University, Semmelweis University 1st Department of Pediatrics, Bokay u. 53-54. H-1083 Budapest, Hungary; 10 Horizon Discovery Ltd, Building 7300, IQ Cambridge, CB25 9TL, UK; 11 Department of Molecular Biology, NMI Natural and Medical Sciences Institute at the University of Tübingen, Markwiesenstrasse 55, 72770 Reutlingen, Germany; 12 Bayer Schering Pharma AG Müllerstraße 178, 13353 Berlin, Germany

## Abstract

The European Union multi-disciplinary Personalised RNA interference to Enhance the Delivery of Individualised Cytotoxic and Targeted therapeutics (PREDICT) consortium has recently initiated a framework to accelerate the development of predictive biomarkers of individual patient response to anti-cancer agents. The consortium focuses on the identification of reliable predictive biomarkers to approved agents with anti-angiogenic activity for which no reliable predictive biomarkers exist: sunitinib, a multi-targeted tyrosine kinase inhibitor and everolimus, a mammalian target of rapamycin (mTOR) pathway inhibitor. Through the analysis of tumor tissue derived from pre-operative renal cell carcinoma (RCC) clinical trials, the PREDICT consortium will use established and novel methods to integrate comprehensive tumor-derived genomic data with personalized tumor-derived small hairpin RNA and high-throughput small interfering RNA screens to identify and validate functionally important genomic or transcriptomic predictive biomarkers of individual drug response in patients. PREDICT's approach to predictive biomarker discovery differs from conventional associative learning approaches, which can be susceptible to the detection of chance associations that lead to overestimation of true clinical accuracy. These methods will identify molecular pathways important for survival and growth of RCC cells and particular targets suitable for therapeutic development. Importantly, our results may enable individualized treatment of RCC, reducing ineffective therapy in drug-resistant disease, leading to improved quality of life and higher cost efficiency, which in turn should broaden patient access to beneficial therapeutics, thereby enhancing clinical outcome and cancer survival. The consortium will also establish and consolidate a European network providing the technological and clinical platform for large-scale functional genomic biomarker discovery. Here we review our current understanding of molecular mechanisms driving resistance to anti-angiogenesis agents, the current limitations of laboratory and clinical trial strategies and how the PREDICT consortium will endeavor to identify a new generation of predictive biomarkers.

## Background

Despite an improved understanding of molecular mechanisms driving distinct cancer cell biological processes, cost-utility analysis of certain targeted therapeutics has raised concerns regarding the ability of health economies to afford such developments [1]. European Health Technology Appraisal committees are struggling to define cost thresholds above which novel agents are no longer affordable, with 90% of cancer drugs approved over the past 4 years costing >13,000 Euros for a 12-week course. The model adopted in the United Kingdom by the National Institute for Health and Clinical Excellence (NICE) is to offer treatment reimbursed by the National Health Service if the cost of therapy is below a threshold of approximately 30,000 to 40,000 Euros per quality adjusted life year (QALY). Drug rationing based on cost/benefit analyses (for example, cost per QALY) has profound implications, particularly for disease subtypes for which limited effective treatments exist, where any gain in quality of life or progression-free survival attributable to a new therapy is regarded as the new gold standard. Such rationing has recently been proposed for anti-angiogenesis agents in renal cell carcinoma (RCC) and other solid tumors where the cost per QALY gained does not meet such stringent thresholds. Such cost-benefit considerations together with the economic climate have precipitated imminent changes to clinical trial design in cancer medicine through the consideration of health economic costs as well as clinical benefit rates [1], mandating the requirement for parallel predictive biomarker discovery approaches.

## PREDICT consortium background

Health economic and clinical trial considerations in renal carcinoma combined with contemporary developments in high-throughput functional genomics biology have led to the unification of six leading European research centers with two SMEs (small and medium sized enterprises) and the Royal Marsden/Institut Gustave Roussy renal cancer biomarker-driven clinical trials network, into the Personalised RNA interference to Enhance the Delivery of Individualised Cytotoxic and Targeted therapeutics (PREDICT) consortium. PREDICT unites world-class clinical trial centers with international leaders in tumor functional genomics and genome-wide sequencing to identify the next generation of individualized predictive biomarkers in cancer medicine. Importantly, this consortium encompasses the largest combined renal cancer patient referral base in Europe that has standardized operating procedures for tissue collection and processing, adhering to common European Good Clinical Practice trial guidelines and ethical principles.

Inter- and intra-tumor molecular heterogeneity has severely limited the ability to define key components of drug response pathways in cancer medicine that might enable the better prediction of patient benefit in advance of treatment exposure. The PREDICT consortium recognizes that the development of personalized treatment approaches adapted to the molecular phenotype of individual tumors will be required to direct therapeutics appropriately and identify novel mechanisms of drug resistance and combination strategies to prolong drug sensitivity.

PREDICT's approach to biomarker discovery differs from conventional associative learning approaches, which can be susceptible to chance associations that lead to overestimation of true clinical accuracy [2,3]. PREDICT's objectives depend on the identification of cancer cell genomic regulators of drug response through the functional annotation of the cancer transcriptome using high-throughput personalized RNA interference (RNAi) techniques integrated with genomics analyses of primary tumor tissue from single-drug clinical trials before and after drug therapy. These methods present a more tractable strategy that is less susceptible to chance associations and may allow the identification of predictive genomics markers of drug response and the identification of consistent molecular pathways mediating therapeutic resistance. This biomedical consortium allows rapid and efficient patient recruitment combined with meticulous tumor tissue processing necessary for biomarker-driven functional genomics approaches to provide more cost-effective personalized therapy with a higher therapeutic index.

## Renal cell carcinoma

PREDICT has identified RCC as a disease lacking predictive biomarkers for the most active therapeutic compounds targeting the mammalian target of rapamycin (mTOR) and vascular endothelial growth factor (VEGF) pathways. About 90% of kidney tumors arise in the renal parenchyma (RCCs) whilst 10% arise in the renal pelvis or ureter (transitional cell carcinomas). RCC is a relatively rare tumor with a rising incidence, accounting for approximately 3% of malignancies in the European Union (EU) with 63,600 cases reported in 2006 [4]; a third to a half of those diagnosed with kidney cancer will die as a consequence of the disease. Of the ten countries in the EU with higher than average incidence rates, seven are former Eastern Bloc countries; the reason for this observation is unknown. RCC is also commoner in men than women for unknown reasons and generally affects those over 60 years of age; as a consequence, the incidence of the disease is anticipated to increase in the future in the EU in the face of an aging population. Individuals affected by RCC may present with symptoms and signs of localized disease, such as loin pain or hematuria but the diagnosis is increasingly made incidentally as a result of imaging performed for unrelated reasons. The mainstay of curative treatment is nephrectomy, and palliative debulking nephrectomy has been shown in randomized studies to result in a survival benefit in fit patients with metastatic disease and is consequently a mainstay of the treatment of RCC.

There are five histological subtypes of RCC: clear cell (75 to 80%), papillary (10 to 15%), medullary, chromophobe and collecting duct (under 5% each). Clear cell histology is associated with dysfunction of the Von Hippel Lindau tumor suppressor gene (*VHL*) in the majority of cases [5]. The product of the *VHL *gene (pVHL) is a component of a ubiquitin ligase complex that mediates the cellular response to hypoxia. In normoxic conditions pVHL binds hypoxia inducible factor (HIF)-1a and HIF2a, leading to ubiquitination and proteasomal degradation. In hypoxic conditions or in the absence of pVHL, HIF1a and HIF2a accumulate and upregulate the production of growth factors such as platelet-derived growth factor (PDGF) and vascular endothelial growth factor (VEGF) at the transcriptional level.

## Recent developments in targeted therapeutics in renal cell carcinoma

Prior to 2006, systemic treatment options for advanced RCC were limited to cytokine-based therapies, such as IL-2 and IFN-α, which are associated with low response rates (typically <20%) and significant toxicity. Since 2006, there have been unprecedented advances in the systemic treatment of advanced RCC and six new drugs have been approved for this indication: the monoclonal anti-VEGF antibody bevacizumab, the multi-targeted tyrosine kinase inhibitors sorafenib, sunitinib and pazopanib, which inhibit VEGFRs, and the mTOR inhibitors everolimus and temsirolimus. Each of these drugs has shown efficacy in RCC in randomized studies in comparison with either placebo or IFN-α [6-10]. Further studies have shown that several other multi-targeted VEGFR kinase inhibitors, such as pazopanib and axitinib, are also active in this disease [11-13]. All of these agents have a putative anti-angiogenic mechanism of action whilst the mTOR inhibitors everolimus and temsirolimus may have direct anti-tumor effects in RCC. mTOR inhibition results in attenuation of VEGFR/phosphatidylinositol-3-kinase (PI3K)/AKT signaling and HIF down-regulation, further supporting a role for these small molecules in the inhibition of angiogenesis.

Despite the fact that clinical trials establishing the activity of these agents in RCC represent landmark studies, between a third and two-thirds of patients (depending on prognostic factors and clinical setting) have intrinsically resistant disease and do not benefit from treatment with agents such as sunitinib or everolimus. Furthermore, all patients develop acquired resistance to therapy and the median progression-free survival in the clinical trials of the most active agents in RCC ranges from 4 to 11 months, indicating the need to identify predictive biomarkers of drug response and identify new targets suitable for therapeutic intervention to delay the acquisition of resistance. The design of these trials in RCC was dictated mainly by clinical considerations and, in general, scientific questions were not addressed. Tumor biopsies were not collected systematically as part of these trials, and although in some cases efforts have been made to obtain archival paraffin-embedded tumor material from the time of nephrectomy, it is rare to obtain material from sufficient numbers of study participants to allow meaningful molecular analysis.

## PREDICT will address anti-angiogenesis research priority areas

Despite advances in the therapeutic management of RCC, there are no established predictive biomarkers of response to these agents in RCC or other solid tumor types, and in excess of 30% of patients will not derive benefit from treatment. PREDICT is focusing on four recently identified research priority areas: first, identification of predictive and surrogate biomarkers, which will help select patients for particular therapies and provide early information on treatment efficacy; second, **d**etermination of the mechanisms of acquired resistance to VEGF-targeted therapy; third, determination of mechanisms of response to current agents, with a particular emphasis on how this might lead to the development of more effective agents and more rational treatment sequencing; and fourth, identification of new targets in RCC. Predictors of response to inhibitors of the VEGFR-mTOR-HIF signaling axis are likely to be relevant to other tumor types in which these agents are active or in which mTOR/HIF signaling is critical [14]. Identification of such factors would allow therapy to be directed to those patients most likely to benefit, promoting clinical and health economic advantages.

## Molecular mechanisms of sunitinib activity and resistance in RCC

VEGF and PDGF are important pro-angiogenic factors driving tumor angiogenesis and increasing tumor vessel stability by activating the endothelial VEGFR [15] and pericyte PDGF receptor (PDGFR) tyrosine kinases, respectively. The persistent upregulation of VEGF and PDGF in the majority of RCCs through inactivation of *VHL *fosters angiogenesis and growth of these tumors. The multitargeted tyrosine kinase inhibitor sunitinib targets the VEGFR, PDGFR, cKIT, FLT3 (FMS-like tyrosine kinase 3), RET and the CSF1 (colony stimulating factor 1) receptor and other tyrosine kinases [16]. Sunitinib has consistently led to decreased intratumoral blood flow based on functional imaging assessment in clinical trials [17] and to anti-angiogenic effects in RCC xenograft mouse models. In contrast, sunitinib has no direct effect on RCC cell line growth *in vitro *[18]. Thus, clinical sunitinib activity in RCC is thought to be a consequence of its anti-angiogenic activity. Several potential mechanisms of resistance to anti-angiogenic drugs like sunitinib have been proposed and two main types of resistance can be distinguished: resistance of the tumor vasculature to the inhibition of VEGF and PDGF signaling (vascular resistance); and resistance of cancer cells to the hypoxic and nutrient-depleted microenvironment induced by anti-angiogenic effects (hypoxia resistance - resistance to the effector mechanism of anti-angiogenic treatment).

### Vascular resistance

Vascular resistance to anti-angiogenic drugs has been shown to occur, amongst others, through activation of alternative pro-angiogenic pathways [19]. For example, increased IL-8 secretion by RCC cells in mouse xenograft models has been found to confer sunitinib resistance *in vivo *and immunohistochemical measurement of IL-8 in patient tumor samples also correlated with clinical sunitinib resistance in a retrospective analysis of a small number of patients [18]. Cancer treatment with anti-angiogenic drugs induces a short period of vascular normalization with improved tumor oxygenation, followed by impaired tumor perfusion leading to increased hypoxia and lack of nutrients [20,21]. Hypoxia is thought to be the predominant effector mechanism of anti-angiogenic drugs because oxygen has a shorter diffusion limit (approximately 150 μm) in tissues than critical nutrients like, for example, glucose [22]. Robust data regarding the severity of hypoxia induced by anti-angiogenic drug treatment are lacking; however, oxygen levels below 0.5% can be found in untreated tumors and are likely to be significantly aggravated by anti-angiogenic treatment. Oxygen concentrations below 0.5% have anti-proliferative effects on many cancer cell lines *in vitro *and can cause apoptosis and necrosis.

### Hypoxia resistance

Hypoxia resistance and inherent tolerability to hypovascular environments have been observed in some cancer types [19]. Furthermore, the selection of hypoxia-resistant cancer cells with the ability to thrive in a therapy-induced low oxygen environment has previously been reported [23] and inactivating p53 mutations have been identified to contribute to hypoxia resistance. A large scale small interfering RNA (siRNA) screen of hypoxia resistance genes in *Caenorhabditis elegans *highlighted the complexity of this process, identifying almost 200 genes from a variety of functional gene groups, such as signaling molecules, metabolic genes and genes controlling protein translation, that influence survival under hypoxic conditions [24]. Knockdown of several of these genes also led to hypoxia resistance in human cancer cell lines. This indicates that hypoxia sensitivity is strongly determined by the genetic background through distinct and complex cellular pathways. Thus, hypoxia resistance is likely to contribute to VEGF-targeted therapeutic resistance [25-27]. Furthermore, hypoxia can induce genetic instability in cancer cells [28], and the steady proliferation of hypoxia-resistant cancer cell clones could foster the acquisition of additional mutations that may permit the tumor to re-establish a resistant vasculature (for example, through activation of alternative pro-angiogenic pathways and factor secretion).

## Molecular mechanisms of everolimus activity and resistance in RCC

The rapamycin-like (rapalog) drug everolimus inhibits the serine/threonine protein kinase mTOR after forming a complex with the intracellular protein FKBP12 (FK506 binding protein-12). mTOR is a component of two distinct cellular multiprotein complexes, mTOR complex (mTORC)1 and mTORC2, and only mTORC1 is directly inhibited by the rapalog-FKBP12 complex [29]. Activation of mTORC1 increases protein translation and promotes entry into the G1 phase of the cell cycle by phosphorylation of downstream substrates, including ribosomal S6 kinase 1 (S6K) and 4EIF binding protein 1 (4EBP1). Inhibition of mTORC1 by everolimus leads to G1 cell cycle arrest, autophagy induction and cytostasis of many RCC cell lines *in vitro*. An important role of the mTOR pathway in clear cell RCC (CCRCC) is supported by the occurrence of these cancers in patients with tuberous sclerosis, who have a constitutively activated mTOR pathway. Phosphorylation of the S6 protein, mediated by S6K activity, an mTOR target, was significantly higher in CCRCC compared to other RCC subtypes and is associated with poorer outcome [30]. The majority of CCRCCs are deficient for the tumor suppressor gene *VHL*, which leads to the upregulation of the transcription factor subunits HIF1a and HIF2a. mTORC1 regulates HIF1a protein translation and thus controls transcription of the downstream target VEGF. Thus, mTORC1 inhibition with rapalogs such as everolimus reduces HIF1a protein levels and decreases VEGF transcription and neo-angiogenesis in xenograft mouse models [31,32]. This effect may contribute to the activity of mTOR inhibitors in patients with *VHL*-deficient RCCs. mTORC1 is also a component of the downstream signaling cascade of the VEGFR in endothelial cells and inhibition by everolimus impairs endothelial proliferation, which amplifies the anti-angiogenic effect. Thus, everolimus is likely to have dual activity in RCCs through direct inhibition of cancer cell proliferation and through the inhibition of tumor angiogenesis.

### Everolimus resistance

Several molecular mechanisms that may be implicated in resistance to rapalog mTOR inhibitors have been documented in laboratory model systems. These include a negative feedback loop from S6K to the insulin receptor substrates (IRS) 1 and 2. Inhibition of mTORC1 and S6K activity by rapalogs can increase IRS1/2 activity, which leads to enhanced Akt phosphorylation in cells where this negative feedback loop is active [33]. The ensuing Akt activation may promote cell survival and proliferation and thus escape from the antitumor activity of everolimus. Rapalog exposure can also indirectly inhibit the assembly of mTORC2, probably by promoting the sequestration of mTOR into mTORC1, which effectively eliminates mTOR from mTORC2 [34,35]. This has been observed in 20% of tested cancer cell lines, and it has been speculated that only tumors responding to rapalogs with mTORC1 and mTORC2 inhibition may be clinically sensitive to this class of agents. Concomitant activation of the Ras-mitogen-activated protein kinase (MAPK) pathway has been found to override rapalog sensitivity in prostate epithelial cells [36] and in melanoma cell lines [37]. Thus, cells with an activated Ras-MAPK pathway may require the combined inhibition of the Ras pathway and of mTOR to overcome resistance to mTOR inhibitors alone. Despite the discovery of these feedback and parallel pathways, their potential role in clinical everolimus resistance and sensitivity is unknown. Immunohistochemical studies of mTOR pathway activity in pre-treatment RCC biopsies from patients receiving the rapalog temsirolimus showed a weak but statistically significant correlation of phosphorylated S6, the substrate of S6K, with clinical response [38]. However, many tumors with highly phosphorylated S6 were refractory to the antiproliferative activity of temsirolimus, indicating that other, hitherto unknown factors play a role. Activation of PI3K-mTOR signaling through PTEN (phosphatase and tensin homologue) inactivation was thought to sensitize tumors to mTOR inhibition, but no correlation of PTEN status and temsirolimus response in RCC was found in the same study [38]. Rapalog suppression of HIF1a-mediated transcriptional activation of pro-angiogenic factors like VEGF may contribute to the activity of rapalogs in CCRCCs. This is supported by the discovery that *VHL *loss and the resulting HIF1a upregulation confers heightened sensitivity to the rapalog temsirolimus in RCC cells [31]. However, responses to temsirolimus can also occur in *VHL*-positive RCCs, indicating that factors determining overall mTOR inhibitor sensitivity or resistance are poorly understood. It is unknown how much direct anti-cancer cell effects and anti-angiogenic everolimus effects contribute to clinical sensitivity. If anti-angiogenic activity predominates, anti-angiogenic resistance mechanisms as outlined for sunitinib (vascular resistance and hypoxia resistance) may play a major role.

The lack of suitable tumor samples from well annotated clinical trials and the resulting reliance on mouse xenograft and *in vitro *cancer models has precluded the identification of clinically relevant predictive biomarkers for mTOR inhibitors and anti-angiogenic drugs in RCC and other solid tumors [39]. Thus, novel and unbiased approaches integrating functional genomics datasets with molecular analyses of human tumor samples using PREDICT consortium validated technologies [40] represent a rational step to identify predictive biomarkers to these agents.

## Pre-operative biomarker-driven RCC clinical trials

The necessity for new biomarker discovery approaches and the need for predictive biomarkers for mTOR inhibitors and VEGF targeted anti-angiogenic therapeutics to improve clinical outcomes and the cost-effectiveness of these drugs in cancer medicine have led the PREDICT consortium to design renal cancer clinical trial endpoints using these agents in parallel with robust tumor genomics, functional genomics and other molecular analyses to accelerate predictive biomarker discovery.

**Figure 1:**
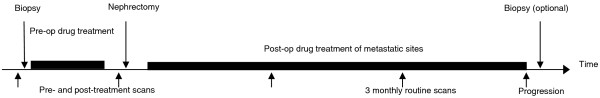
**Overview of the PREDICT neo-adjuvant clinical trial strategy**.

In order to identify the next generation of predictive biomarkers, we have designed clinical trials specifically to include the collection of fresh tissue to synergize with parallel high-throughput genomics analyses (Figure 1). Two such clinical trials, E-PREDICT [41] and S-PREDICT/PREINSUT [42], have been initiated and are currently recruiting patients. Fresh tissue will be collected in a quality-controlled setting before and after drug therapy for molecular analyses that can be correlated with clinical efficacy. Each of these pre-nephrectomy RCC clinical trials using the mTOR inhibitor everolimus (E-PREDICT) and the VEGFR targeted therapeutic sunitinib (S-PREDICT/PREINSUT) will recruit 60 patients in discovery cohorts and 60 patients in validation cohorts for predictive biomarker validation.

Study participants have metastatic RCC, and palliative nephrectomy has been recommended as part of routine clinical management. In the PREDICT trials, tumor biopsies are taken and sunitinib or everolimus administered in a 'window of opportunity' before nephrectomy. The therapeutic agent is stopped 1 to 2 weeks before nephrectomy for safety and scientific reasons and restarted after nephrectomy until the eventual development of progressive disease in metastatic lesions. The scientific reason for stopping drug 1 to 2 weeks before nephrectomy is so that the acute transcriptional effects of drug exposure are limited. Response to treatment will be assessed at primary and metastatic sites using the Response Evaluation Criteria In Solid Tumors (RECIST) by computed tomography (CT) imaging before treatment initiation and after exposure to the therapeutic agent before patients undergo nephrectomy. Further imaging of metastatic sites will be performed after nephrectomy at 3-monthly intervals; efficacy data will be available for all patients based on evaluation of metastatic sites.

## PREDICT integrative genomics developments guiding biomarker discovery in cancer medicine

Approaches used by PREDICT consortium members have been designed to avoid or overcome the various pitfalls of high-throughput associative studies of gene expression datasets [3,43] in order to develop the next generation of prognostic and predictive biomarkers. The potential to rapidly identify predictive biomarkers of drug response in tumor tissue to define sensitive and resistant patient cohorts has recently been accelerated through advances in functional genomics techniques that have been intensively developed by the PREDICT consortium using large scale RNAi screening approaches [44-47].

Through the use of this technology, the consortium has identified genes regulating response and resistance to common cytotoxic agents used in cancer medicine [40,46,48-50]. Through the integrative genomics analysis of these functional RNAi datasets in breast and ovarian cancer, we have identified regulators of mitotic arrest and ceramide metabolism as mediators of taxane resistance and confirmed their relevance in clinical trial cohorts [40,46,48-50]. For example, silencing of the ceramide transporter CERT was shown to confer sensitivity to paclitaxel across multiple cancer cell lines and follow-up analysis revealed that CERT was overexpressed in two separate paclitaxel-resistant cell lines. Analysis of microarray expression data from the OV-01 clinical trial revealed that over-expression of CERT occurred in ovarian cancers from patients with paclitaxel resistant disease, suggesting a role for this gene product in the regulation of response to paclitaxel *in vivo *[46].

Successful integration of RNAi functional genomics screening results with tumor gene expression data in order to identify a predictor of neoadjuvant paclitaxel response in breast cancer was dependent on the identification of gene coexpression modules representative of mitotic arrest and ceramide metabolic pathways relevant to drug response. The combination of these modules into a 'functional metagene' shows promise as a paclitaxel-specific predictive biomarker [40] that is predictive of pathological complete response to paclitaxel in breast cancer with a high sensitivity and specificity (area under the receiver operating characteristic curve (AUC) = 0.8) [40], outperforming any other clinical or molecular predictor of paclitaxel sensitivity identified to date.

Further supporting integrative genomics approaches to the identification of novel drug response mechanisms *in vivo*, we have integrated complex cancer datasets (gene expression and copy number data) to identify a particular chromosomal region that contributes to anthracycline resistance when amplified in breast cancer. Two causative genes, *LAPTM4B *and *YWHAZ*, were identified from this region: one is a known anti-apoptosis gene, and one is a novel gene affecting drug transport. These genes are strongly predictive of anthracycline resistance, and rigorous clinical evaluation is ongoing [51].

We have also demonstrated that molecular hypotheses can be utilized to predict drug response *in vivo*. We formed a rational hypothesis about drug mechanism to suggest a predictor of response to cisplatin. Briefly, we noticed links between *BRCA1 *mutations, cisplatin sensitivity, and DNA repair pathway competence. We developed a SNP array-based surrogate marker of DNA repair pathway competence and found that it strongly predicted for neoadjuvant cisplatin pathological complete response in a small cohort of estrogen receptor-negative/progesterone receptor-negative/ERBB2-negative breast cancer patients [52]. We have derived a robust gene expression signature of chromosomal instability, which is prognostic in several types of solid tumor [53] and predictive of paclitaxel resistance in ovarian cancer [50]. We have also identified a blood-based gene expression biomarker of early-stage Parkinson's disease [54], which is currently being validated in a larger study, and have integrated diverse genomic data sets to generate an atlas of disease-associated protein complexes, several of which were novel [55].

These studies highlight the power of comprehensive functional genomics datasets combined with monotherapy clinical trial tumor genomics datasets to illuminate the clinical relevance of specific genes to individual patient drug sensitivity. Furthermore, the studies provide robust and efficient methodological tools to accelerate predictive biomarker development and identify mechanisms of drug resistance that will be applied to biomarker discovery in RCC in this proposal.

## PREDICT technologies for biomarker discovery in RCC

### PREDICT RNA interference screens

Based on the clinical and molecular evidence reviewed above, we hypothesize that resistance of RCCs to sunitinib and everolimus might occur through one or a combination of the mechanisms (Table 1). PREDICT consortium's functional genomics RNAi approaches will be applied to identify genes contributing to these resistance mechanisms.

**Table 1:** Mechanisms of resistance to sunitinib and everolimus

**Potential mechanisms of sunitinib resistance**

Hypoxia resistance of RCC cells
Vascular resistance to VEGFR and PDGFR inhibition by sunitinib

**Potential mechanisms of everolimus resistance**
Resistance of RCC cells to direct anti-proliferative everolimus effects
Resistance of HIF1α target gene expression to repression by everolimus
Hypoxia resistance of RCC cells
Vascular resistance to VEGF pathway inhibition

PDGFR, platelet derived growth factor receptor; RCC, renal cell carcinoma; VEGF, vascular endothelial growth factor; VEGFR, VEGF receptor.

The PREDICT consortium will use novel small hairpin RNA (shRNA) and siRNA screening approaches to identify genes consistently regulating response to hypoxia and everolimus exposure in multiple renal cancer cell lines propagated from tumors *ex vivo*. Consistent with PREDICT's recently published predictive biomarker in breast cancer based on this strategy, genes identified across multiple cell lines or in multiple screens that promote sensitivity or resistance to hypoxia or everolimus exposure may be implicated in everolimus and sunitinib sensitivity in patients. Central to this proposal will be the derivation of up to 30 *ex vivo *cultured patient-derived RCC cell bulks from which personalized RNAi (personalized shRNA) libraries will be generated to identify tumor-individualized autologous mechanisms of drug response. These will be used to yield vital information, complementary to the unbiased siRNA and shRNA screening approaches about the functional role of each gene expressed in tumor samples that may determine resistance or sensitivity to sunitinib or everolimus.

### PREDICT tumor genomics analysis

PREDICT has focused on standardizing tissue collection procedures across clinical sites involved in the S-PREDICT/PREINSUT and E-PREDICT clinical trials. RNA and DNA extracted from microdissected cancer cells from pre- and post-treatment specimens will be hybridized to gene expression and DNA SNP/comparative genomic hybridization arrays, respectively. A kinome activity assay will evaluate the activity of 267 kinases (160 serine threonine kinases and 107 tyrosine kinases) on tumor samples following treatment in order to identify molecular pathways regulated following everolimus and sunitinib exposure in resistant and sensitive disease.

### PREDICT tumor exome-sequencing analysis

Nephrectomy samples (and corresponding matched pre-treatment tumors and germline DNA) from patients with progressive/resistant disease will be available for whole-genome exon sequencing to identify candidate genes associated with everolimus or sunitinib resistance *in vivo*. Tumors from patients with imaging-defined drug-resistant disease within these clinical trials will be subject to exon-capture sequencing before and after drug exposure to characterize the somatic mutational spectrum in resistant tumors [56]. Directed sequencing methods will be used to confirm the specificity of these somatic mutations to drug-resistant compared to drug-sensitive tumors.

### PREDICT *ex vivo *renal cancer cell line culture

Renal cancer xenograft and cell culture models are being established from surgical specimens to support the personalized identification of novel biomarkers in RCC. Surgically resected tumor specimens are transplanted subcutaneously into immunodeficient NOD/SCID mice, and following successful engraftment (>30% success rate expected), parts of the tumor material will be used for further passage, cryopreservation (tumor bank) and to establish *ex vivo *propagation of cancer cell lines for use in the functional genomics personalized shRNA screens.

### PREDICT integrative genomics analysis

**Figure 2:**
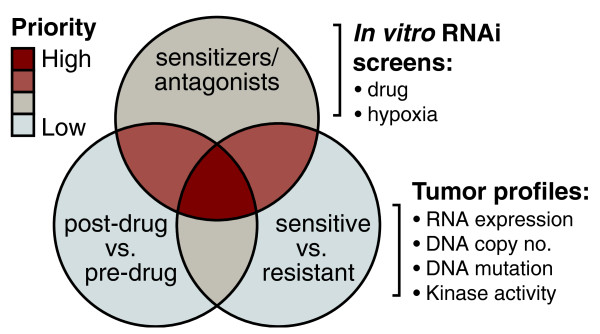
**Prioritization of predictive biomarkers for validation**.

Data from the unbiased and personalized RNAi screens will be integrated with genomics and proteomics datasets from patient tumors from the discovery phase of the two clinical trial cohorts and genes that are identified through multiple approaches (for example, modifying resistance in functional genomic screens and altered expression/copy number/sequence in resistant versus sensitive tumors; Figure 2) will be prioritized for development of predictive signatures of sunitinib and everolimus response for assessment in the validation phases of the two clinical trials.

Genomic, proteomic and functional RNAi datasets will be integrated together with clinical response data into a meta-dataset containing expression, mutation, copy number and functional data in a genome-wide manner. Bioinformatics analysis of the meta-dataset for genes altered in resistant versus sensitive samples that functionally influence resistance in laboratory model systems of RCC will lead to the prioritization of predictive biomarkers for sunitinib and everolimus in the validation cohorts of the E-PREDICT and S-PREDICT/PREINSUT clinical trials (Figure 2). Through this approach, the consortium will meet its overall objectives of identifying robust predictors of response to anti-angiogenic therapies. More importantly, the clinical trial and functional genomics framework will be established to enable the rapid development of the next generation of predictive biomarkers across a wide range of solid tumor types.

## Integration of personalized functional genomics into the clinical setting

The generation of personalized RNAi screening approaches, representing the complete transcriptome of distinct tumors from individual patients, allows the identification of genes that are differentially expressed in tumors that impact upon drug response. Importantly, such an approach, if validated in RCC, would be directly applicable to other tumor types for which tumor biopsies could be acquired prior to treatment exposure within defined single-drug clinical trials. This approach may allow an unprecedented opportunity to identify patient-specific drug sensitivity pathways in cancer medicine and may precipitate improvements to clinical trial design and the stratification of patients according to defined personalized biomarkers of drug response. Importantly, this cost-effective technique is aimed at reducing health economic costs and improving patient quality of life due to the specific application of novel therapeutics specifically to patients with drug-sensitive disease.

## Conclusions

The health economics of targeted therapeutic strategies with benefit confined to distinct but unknown patient subpopulations has major implications for future drug development, for the provision of affordable healthcare to all individuals within the EU, and for patient access to therapies that will genuinely offer therapeutic benefit to a minority of patients. Indeed, in a recent analysis of patient survival for all cancers across Europe, it was recognized that in the future as oncology costs continue to escalate, the best treatments will only be available to the wealthiest, as member states conclude that resources cannot be allocated to provide optimal cancer care for all patients. In this publication, the urgent need for a radical evaluation of cost considerations in cancer research and the requirement for investment in new technology was recognized [57]. A solution to these problems is to rapidly identify predictive molecular biomarkers of drug response, to limit patient exposure to costly and ineffective therapies whilst targeting sensitive patient cohorts, using integrative genomics methods and standardized clinical trial infrastructure. These methods will be applicable to biomarker discovery efforts across all cancer types and therapeutic modalities for which no predictive assays exist.

Through the identification of genes functionally required for everolimus and sunitinib response integrated with parallel whole-genome analysis of clinical trial tissue, we will identify robust and validated genomics markers to predict therapeutic outcome. Through these approaches we hope to ultimately reduce the cost per QALY associated with drug treatment, allowing wider access to active agents in sensitive patient cohorts.

## Abbreviations

CCRCC: clear cell renal cell carcinoma; EU: European Union; HIF: hypoxia inducible factor; IL: interleukin; IFN: interferon; IRS: insulin receptor substrate; MAPK: mitogen-activated protein kinase; mTOR: mammalian target of rapamycin; mTORC: mammalian target of rapamycin complex; PDGF: platelet derived growth factor; PDGFR: platelet derived growth factor receptor; PI3K: phosphatidylinositol-3-kinase; PREDICT: Personalised RNA interference to Enhance the Delivery of Individualised Cytotoxic and Targeted therapeutics; PTEN: phosphatase and tensin homologue; QALY: quality adjusted life year; RCC: renal cell carcinoma; RNAi: RNA interference; S6K: S6 kinase; shRNA: small hairpin RNA; siRNA: small interfering RNA; SNP: single-nucleotide polymorphism; VEGF: vascular endothelial growth factor; VEGFR: vascular endothelial growth factor receptor; VHL: Von Hippel-Lindau.

## Competing interests

JML has accepted honoraria and grants from both Novartis and Pfizer, SO and BE have accepted honoraria from both Novartis and Pfizer. CS receives clinical translational trial grant funding from Novartis. Pfizer and Novartis are providing trials funding for S-PREDICT/PREINSUT and E-PREDICT, respectively. The other authors declare that they have no competing interests.

## Authors' contributions

CS, JML, MG, AE, KAB, BN and ZS wrote the manuscript. CS and ZS conceived the scientific approach, JL and SO designed the clinical trials. All authors reviewed the manuscript.
